# Active Parental Mediation and Adolescent Problematic Internet Use: The Mediating Role of Parent–Child Relationships and Hiding Online Behavior

**DOI:** 10.3390/bs13080679

**Published:** 2023-08-12

**Authors:** Jingjing Liu, Li Wu, Xiaojun Sun, Xuqing Bai, Changying Duan

**Affiliations:** 1 School of Psychology, Central China Normal University, Wuhan 430079, China; ljj2021@mails.ccnu.edu.cn (J.L.); bxq1999@163.com (X.B.); dcy-2018@mails.ccnu.edu.cn (C.D.); 2 Department of Students’ Affairs, Yancheng Teachers University, Yancheng 224007, China; 3 Key Laboratory of Adolescent Cyberpsychology and Behavior (CCNU), Ministry of Education, Wuhan 430079, China; 4 School of Education, Central China Normal University, Wuhan 430079, China; wuli1030@ccnu.edu.cn; 5 Beijing Normal University Collaboration Innovation Center, Central China Normal University Branch, Wuhan 430079, China

**Keywords:** adolescents, parental active mediation, parent–child relationship, hiding online behavior, PIU

## Abstract

In today’s information society, with the growing integration of the Internet into individuals’ lives, problematic Internet use (PIU) among adolescents has become more prevalent. Therefore, we conducted this study to investigate the correlation between active parental mediation and PIU in adolescents, as well as the potential mediating roles of parent–child relationship and adolescents’ hiding online behavior. A total of 539 middle school students (mean age = 13.384) were recruited for this study and participated by completing a series of paper-and-pencil questionnaires. The findings indicated a significant negative relationship between active parental mediation and PIU. Furthermore, both the mediating role of the parent–child relationship and the role of hiding online behavior were found to be significant. Specifically, the mediating role is comprised of two paths: the independent mediating role of the parent–child relationship, and the sequential mediating role involving both the parent–child relationship and hiding online behavior. The study contributes an innovative theoretical perspective to deepen the understanding of the formation mechanism of PIU. Moreover, it offers practical empirical insights for the prevention and intervention of PIU among adolescents.

## 1. Introduction

In contemporary information-based society, the Internet has deeply infiltrated into people’s daily lives, offering numerous conveniences to individuals. It serves as a facilitator for social connectivity, a source of valuable information, and a platform for entertainment [1]. Despite moderate Internet usage being beneficial to the development of children and adolescents, excessive Internet use, problematic Internet use (PIU), or Internet addiction can have detrimental effects. PIU is commonly defined as the excessive preoccupation with and the loss of control over Internet usage, leading to adverse consequences in both personal and professional domains [2]. There have been significant research achievements regarding the manifestation of PIU in adolescents and its influencing factors.

### 1.1. PIU and Its Influence among Adolescents

Given the physical and psychological developmental characteristics of adolescents, PIU tends to be more prevalent among adolescents [3]. The prevalence of PIU is rapidly increasing in this group [4]. Particularly during the COVID-19 epidemic, there has been a significant increase in Internet usage among adolescents [5,6,7]. PIU can have various negative effects on adolescents’ developmental adaptation, including sleep disorders [8], academic performance decline [9], low self-efficacy [10], and subsequent impacts on peer relationships and life satisfaction [11], among others. Regarding potential confounding variables or the formation process of PIU, existing research mainly focuses on individual traits [12] and pre-existing environmental factors [13,14]. There are still research gaps concerning how adolescents manage external mediation and online skills. Specifically, it is unclear whether parental mediation can decrease the probability of adolescents employing coping strategies, thereby reducing the likelihood of problematic Internet use. Given their age, adolescents possess a psychological inclination to assert their independence and often possess technological skills that rival or exceed those of their parents. Due to the continuous enhancement of adolescent autonomy, adolescents are unlikely to acquiesce to parental mediation with the same obedience displayed during early childhood. Therefore, when evaluating the actual impact of parental mediation, it is crucial to consider the agency of adolescents.

### 1.2. PIU and Parental Internet Mediation

The family plays a significant role as an environmental factor in influencing various aspects of adolescents’ adaptation, including PIU. With the integration of the Internet into the family system, parental education expands to the online realm, known as parental Internet mediation. Parental Internet mediation entails the activities and strategies employed by parents to guide and regulate their children’s online engagement, as well as to safeguard them from online risks, with the aim of maximizing the positive impacts of the Internet and minimizing the negative effects [15,16]. It can be divided into active (explaining media content to children and communicating educational and critical views and opinions) and restrictive (limiting the content and time of media use). Both active and restrictive mediation can help reduce online risks [17,18,19]. Active parental mediation can improve adolescents’ online literacy [20] and promote positive adaptation [21,22,23], thus receiving more referrals from researchers.

Active mediation is widely recognized as an effective strategy for Internet supervision that helps mitigate the risk of PIU or online risky behavior. Parental mediation characterized by an autonomy-supportive style has been linked to lower levels of internalization problems (such as anxiety, depression) among adolescents, as opposed to mediation with a controlling style [24]. Active mediation or instructive mediation based on parent–adolescent communication has shown superior effectiveness in reducing information disclosure [25] and media-related risks [21] among adolescents. There is a close relationship between online risk behavior and PIU. Therefore, we speculate that active parental mediation is negatively correlated with PIU among adolescents.

### 1.3. The Mediating Role of the Parent–Child Relationship

The parent–child relationship pertains to the interaction between parents and their children. It represents the earliest and most enduring interpersonal relationship that individuals experience throughout their lives [26].

The quality of parent–child relationships influences the developmental adjustment of children and adolescents. A positive parent–child relationship can assist adolescents in handling difficult and stressful events, as those who have a strong parent–child bond exhibit greater emotional resilience and a quicker recovery from negative emotions [27]. Additionally, parent–child relationships are strongly linked to Internet-related problem behaviors. Research studies have discovered that dysfunctional parent–child relationships prompt adolescents to use the Internet more frequently as a means to escape the tension and isolation stemming from problematic interpersonal relationships [28]. On the one hand, PIU may serve as a coping strategy for dealing with emotional difficulties and compensating for the lack of contact with parents [29]. On the other hand, in cases where parents are unsupportive or insensitive, adolescents may employ manipulative strategies to seek parental attention, potentially resulting in the development of PIU [30].

Moreover, the parent–child relationship can be influenced by family interactions, particularly parental mediation practice. Positive parent–child interactions and effective mediation behaviors can foster the development of a strong parent–child relationship, while inappropriate and abusive mediation behaviors can impede the establishment of a healthy parent–child relationship [31]. This holds true for mediation of Internet use as well [32]. Active parental mediation, characterized by a respectful, understanding, and instructive approach, can facilitate communication and interaction between parents and children regarding Internet usage. This, in turn, helps mitigate parent–child conflicts related to Internet use and fosters the maintenance and development of a positive parent–child relationship [33]. Thus, active mediation may enhance parent–child relationship and, in turn, reduce the risk of PIU. Furthermore, research has found that parent–child relationships played a mediating role between active parental mediation and problematic smartphone use [34].

The ecological techno-microsystem theory, derived from the ecological systems theory, provides an ecological perspective when considering the influence of computers, Internet, and other electronic media on children’s development. This perspective emphasizes the dynamic nature of child development and highlights the significance of considering the contextual factors within which children are maturing [35]. Within this framework, the family functions as a vital ecological techno-microsystem. Family cohesion, parental Internet usage, and parental interventions related to adolescent Internet usage, such as online communication between parents and children, hold sway over adolescents’ Internet behaviors. Because the online activities of teenagers have become important aspects of communication and interaction between parents and children, the quality of communication also affects the parent–child relationship. Thus, we propose that the parent–child relationship may serve as a mediator in the connection between parental active mediation and PIU.

### 1.4. The Mediating Role of Hiding Online Behavior

The concept of hiding online behavior from parents currently lacks a clear definition in the academic field. Nevertheless, drawing from existing surveys and their characteristics [36], we define it as a series of online actions by adolescents aimed at evading parental intervention and discipline. According to a survey conducted by McAfee (2012) [36], 70% of teenagers hide online behavior from their parents. Metzger et al. [37] categorized risky Internet use as one of the activities that leads to secrecy among adolescents, in addition to traditional substance use and problematic peer interactions. Engaging in secret-keeping behavior with parents carries inherent risks and is linked to negative outcomes, including low self-esteem and the experience of negative emotions [38,39], external behavioral problems, such as heightened aggression and deviant behavior [40,41]. Building upon this understanding, the present study posits a positive association between hiding online behavior and PIU.

Moreover, hiding online behavior maybe intricately linked to the family environment and its associated parenting styles. Parental support and emotional comfort can positively influence adolescents, fostering a greater inclination towards self-disclosure while diminishing their propensity for confidentiality [42]. Building upon this notion, the present study posits that hiding online behavior from parents potentially mediates the relationship between active parental mediation and adolescents’ PIU.

Additionally, the parent–child relationship is closely intertwined with the act of keeping secrets from parents. Adolescents who have a strained or inadequate parent–child relationship are more inclined to withhold information from their parents [38]. Conversely, the present study postulates a sequential mediating role of the parent–child relationship and the act of hiding online behavior from parents in the context of active parental mediation and PIU.

### 1.5. The Current Study

In summary, the present study investigated the impact of active parental mediation on adolescents’ PIU, while also considering the mediating influence of the parent–child relationship and adolescents’ hiding online behavior from their parents within this association. The study was conducted among Chinese secondary school students, ranging from 12 to 16 years of age. Drawing upon prior research, we posited the following hypotheses:

**Hypothesis 1 (H1).**  *Active parental mediation is negatively related to PIU among adolescents.*

**Hypothesis 2 (H2).**  *Parent–child relationships are positively related to active parental mediation and negatively related to adolescents’ PIU*.

**Hypothesis 3 (H3).**  *Hiding online behavior from parents is negatively related to parent–child relationships and positively related to PIU*.

**Hypothesis 4 (H4).**  *Parent–child relationships mediate the relationship between active parental mediation and adolescent PIU*.

**Hypothesis 5 (H5).**  *Hiding online behavior from parents mediates the relationship between active parental mediation and PIU in adolescents.*

## 2. Materials and Methods

### 2.1. Research Design and Participants

The present study adopts a cross-sectional design. School teachers received brief training in instructing students to complete the questionnaire and addressed any queries they had. Participants took part in the study by completing a paper-and-pencil survey administered in the classroom during school hours, while also providing demographic information. The questionnaires were collected on the spot.

Adolescents were recruited from two middle schools in eastern China using convenience sampling methodology. Out of the total 626 students who volunteered to partake in this study, 539 questionnaires were considered valid after eliminating invalid responses containing incomplete demographic information, non-response for over 20% of survey items, and selecting the same options for all items. This resulted in a valid recall rate of 86.102%. There were 194 (35.992%), 177 (32.839%), and 168 (31.169%) participants in grades 7, 8, and 9, respectively. Of these participants, 277 (51.391%) were male, indicating an equitable distribution across gender and grade levels without significant bias. The participants had a mean age of 13.384 (SD = 0.976).

### 2.2. Measures

#### 2.2.1. Demographics Questionnaire

This section comprised questions regarding the participant’s grade, gender, date of birth, and residential status.

#### 2.2.2. The Problematic and Risky Internet Use Screening Scale (PRIUSS)

This scale was developed by L. A. Jelenchick (2014) [43]. The scale is a theoretically grounded and psychometrically validated tool to assess PIU, specifically designed for adolescents. The scale consists of 18 items and encompasses three sub-dimensions: social consequences (6 items), which describe difficulties in offline communication, forming and maintaining relationships due to Internet use; emotional consequences (5 items), which describe adverse emotions associated with Internet use; and risky and impulsive Internet use (7 items), which describe the inability to control Internet use and the impact on daily life. Example questions included: “How frequently do you opt for online socializing over in-person interactions?”, “How frequently do you experience irritation when unable to access the Internet?”, and “How frequently do you experience sleep disturbances due to nighttime Internet use?”. Participants were requested to indicate their responses based on their Internet usage within the preceding six months. The authors of this study translated the questions into Chinese. The back translations method was employed to ensure accuracy, with an English-majoring researcher, who was not involved in the initial translation, responsible for performing this task. The study utilized a 5-point Likert scale (1 = never, 5 = always). The mean score of the scale was calculated, with higher scores reflecting a greater degree of PIU. In this study, the scale demonstrated strong internal consistency with an overall Cronbach’s α of 0.949. Additionally, the sub-dimensions of social consequences, emotional consequences, and risky and impulsive Internet use yielded respective Cronbach’s α values of 0.860, 0.941, and 0.905. The results of the validation factor analysis were: *χ*^2^/*df* = 6.095, RMSEA = 0.097, CFI = 0.916, TLI = 0.902, and SRMR = 0.059. These findings indicate that the Chinese version of the scale demonstrated favorable levels of reliability, validity, and overall fit.

#### 2.2.3. Active Parental Mediation Scale

The study employed the parental mediation scale developed by Sasson and Mesch (2014) [44], which was adapted by Yao et al. (2022) [45]. The scale comprises two dimensions: active parental mediation and restricted parental mediation. Each dimension consists of six items that inquire about whether participants receive help or guidance from their parents during Internet use. The example question was: “Do your parents assist you when you browse the Internet or search for necessary information?”. The study utilized the active parental mediation subscale. Yao’s study [45] demonstrated the good reliability and validity of the scale in the Chinese edition. Each item offered two response options: “yes” and “no”, with “yes” coded as 1 and “no” as 0. The total score of the scale was calculated, with higher scores indicating a greater degree of active mediation. In this study, the Cronbach’sαfor the active parental mediation scale was 0.895.

#### 2.2.4. Parent–Child Relationship Questionnaire

The study employed the parent–child relationship questionnaire developed by Stattin and Kerr [46], which was translated and modified into a Chinese version by Su et al. [47]. The questionnaire consists of 16 items, with 8 items each measuring the father–child relationship and the mother–child relationship. Participants were asked to reflect upon their relationship with their father/mother during the preceding 6 months (e.g., “Are you disappointed in your father?”). The study employed a 3-point scale with labeled responses: 1 = “never”, 2 = “sometimes”, and 3 = “often”. The questionnaire comprised four reverse questions that were scored using reverse scoring. The mean score of the scale was calculated, and higher score on the scale indicates a stronger parent–child relationship. In Su’s study [47], the Chinese version of the questionnaire demonstrated good reliability and validity. The Cronbach’s α coefficient for the questionnaire in this study was 0.859.

#### 2.2.5. Hiding Online Behavior from Parents Questionnaire

The study utilized a 10-item questionnaire to measure adolescents’ hiding online behavior from parents. The questionnaire was adapted from the 2012 McAfee survey [36], which identified the 10 most commonly employed strategies adolescents use to hide their online behavior from parents. The authors made slight modifications to adapt the questionnaire to Chinese culture when translating it into Chinese. These modifications primarily focused on the online platform Chinese adolescents used. Reverse translation was also carried out to ensure the accuracy of the expression. Participants were queried about the frequency of their engagement in specific behaviors (e.g., closing/minimizing the browser when a parent walked in) while accessing the Internet at home over the preceding 6 months. A 5-point Likert scale, ranging from 1 (never) to 5 (always), was utilized. The mean score of the scale was calculated, and higher scores indicated greater hiding online behaviors from parents. The overall Cronbach’s α coefficient for this scale in the present study was 0.867.

### 2.3. Data Collection

This study received approval from the Academic Committee for Scientific Research at the authors’ institution (IRB NO. 202209013). Prior to the survey, informed consent was obtained from the participating teachers and students.

### 2.4. Statistical Analysis

The data were inputted and analyzed using Epidata 3.1, Mplus 8.0, and SPSS 27.0 software. The average interpolation method was utilized to handle missing values.

Common method deviation test. Heterogeneity of the variables in the questionnaire was examined using Harman’s one-way test to assess common method deviation. The findings revealed that 12 factors had characteristic roots greater than one. The cumulative variance explained by the first factor was 25.875%, which is below the threshold of 40%. Thus, there is no significant common method bias present in this study [48].

The raw data for this article will be made available to the authors upon a reasonable request.

## 3. Results

### 3.1. Preliminary Correlation Analysis

In order to investigate the association between parental mediation and problematic Internet use as hypothesized, we performed a Pearson correlation analysis on the dataset. Age and gender were included as control variables due to their previously observed influence on active parental mediation and adolescents’ PIU. Pearson correlation analyses were conducted for four variables: active parental mediation, parent–child relationship, adolescent hiding online behavior, and PIU. Descriptive statistics and correlation results can be found in Table 1.

Significant correlations were observed among all primary variables. Specifically, active parental mediation showed a significant negative correlation with PIU, indicating that greater parental mediation is associated with a reduced likelihood of problematic Internet use. Therefore, Hypothesis 1 was supported.

Hypothesis 2 examines the relationship between parent–child relationships and active parental mediation, PIU. As shown in Table 1, the parent–child relationships exhibited a positive correlation with active parental mediation, while a negative association was found between the parent–child relationships and adolescents’ PIU. These findings indicate that Chinese adolescents who perceive stronger parental active mediation during Internet use tend to have a better parent–child relationship. Additionally, a stronger parent–child relationship is linked to a reduced likelihood of PIU. Therefore, Hypothesis 2 has been substantiated.

Hypothesis 3 explores the association between hiding online behavior and parent–child relationships, PIU. Based on Table 1, hiding online behavior exhibited a negative correlation with the parent–child relationship and a positive correlation with PIU. Therefore, Hypothesis 3 was supported. Moreover, a notably strong relationship emerged between online hiding behavior and PIU (*r* = 0.545, *p* < 0.001). The correlation among these variables was statistically significant at a high level (*p* < 0.001), allowing for further path analysis to be conducted.

### 3.2. Intermediation Effect Test

Hypotheses 4 and 5 investigated the mediating effects of the parent–child relationship and online hiding behavior. Complex scenarios in studies necessitate the inclusion of multiple mediating variables, enabling a clear elucidation of the independent variable’s impact on the dependent variable [49] (pp. 103–126). Consequently, employing multiple mediation models becomes imperative. Hayes (2009) [50] categorizes these models into single-step multiple mediation models and multi-step multiple mediation models, depending on whether there are interactions among the mediating variables. The single-step multiple mediation model, alternatively referred to as the parallel multiple mediation model, assumes no interaction among the mediation variables. Conversely, the multi-step multiple mediation model, known as the chain multiple mediation model, postulates a hierarchical relationship among the mediation variables, forming a sequential chain of mediators [51]. In this study, a noteworthy negative correlation emerged between the parent–child relationship and hiding online behavior from parents. Consequently, the inclusion of these two mediating variables led us to examine a sequential chain mediating pathway connecting active parental mediation and PIU.

We employed the Hayes PROCESS model 6in SPSS to validate the intermediary path [52]. The percentile bootstrap method was used for bias correction, with repeated sampling 5000 times and calculated 95% confidence interval. Gender and age were included as control variables in the present study. The product of coefficients was tested by arranging them in ascending order, where the 2.5th and 97.5th percentiles formed a 95% confidence interval for ab. If the confidence interval did not encompass 0, the product of coefficients was considered significant [53].

Table 2 demonstrates the findings derived from the regression analysis. It provides an overview of the total indirect effects of the variables on problematic Internet use, as indicated by the *β* coefficient of −0.225, which was found to be statistically significant (*p* < 0.001). The model presented in the table identifies several mediation relationships between predictor variables and problematic Internet use, each displaying varying levels of significance. Importantly, upon the inclusion of mediating variables, the direct path between active regulation and PIU was not significant(*β* = −0.015, *p* > 0.05).

The findings of the Bootstrap mediation test are presented in Table 3. Following the established criteria for the Bootstrap mediation test, the presence of a mediating effect is indicated when the Bootstrap 95% confidence interval does not encompass 0. In line with this criterion, the analysis uncovered that the mediating effect of the parent–child relationship and hiding online behavior was evident in the relationship between active parental mediation and PIU. The mediating effect value was determined to be −0.209, accounting for a substantial proportion of the total effect (−0.225), specifically 93.274%.Specifically, two of the three indirect effect paths were verified, the effect of active regulation on PIU through parent–child relationships and the chain mediation of active regulation on PIU through two mediating variables: parent–child relationships and hiding online behavior. The indirect effects of mediated the two paths accounted for 47.795% and 26.102% of the total effect, respectively.

## 4. Discussion

The present study offers novel insights into the correlation between parental mediation and PIU in China adolescents. Increasing our knowledge about the influence of parental mediation on adolescents across diverse cultures and global locations is vital for fostering a comprehensive understanding. In this regard, it becomes crucial to contextualize parent–child relationships in China, providing a baseline for comprehending the formation process of Internet use within this specific cultural context.

While active parental mediation is of great anticipation, solely investigating the direct association between the two variables may disregard the impact of other potential factors. Therefore, this study aims to comprehensively explore the various pathways through which active parental mediation may exert its influence on adolescents’ general PIU in China. Specifically, it seeks to examine the mediating role played by both the parent–child relationship and adolescents’ tendency to hide online behaviors from their parents.

### 4.1. The Direct Effect of Active Parental Mediation

Consistent with prior research [54,55], the present study corroborates the significant inverse relationship between active parental mediation and PIU among adolescents.

Adolescence is characterized by a quest for autonomy and liberation from parental authority, often accompanied by notable advancements in thinking and cognitive abilities. However, during this period, self-control and cognitive capacities have yet to reach full maturity [56,57]. Consequently, despite engaging in active technology usage to explore the world, construct personal identities, and establish social networks, adolescents face an elevated risk of PIU due to insufficient coping mechanisms [58]. Active parental mediation is a family education practice that entails parents taking an active and proactive role in guiding their children to use the Internet in a rational and secure manner. This involves engaging in discussions with their children to establish ground rules, fostering open verbal communication between parents and adolescents [59]. Therefore, it is advantageous for adolescents to comprehend and embrace their parents’ values regarding the Internet. It also aids parents in understanding the real extent of their adolescents’ Internet usage, enabling them to deliver more focused and effective guidance and assistance. These efforts ultimately contribute to the formation of positive Internet usage habits and the avoidance of problematic online behaviors in adolescents. The ecological techno-subsystem represents a subsystem within the broader ecological system of child development, reflecting the influence of electronic device usage in the family, school, and community on children’s development [60]. Active mediation style, combined with higher levels of media literacy, was associated with a higher likelihood of parents engaging in conversations with their children [61]. The family functions as a direct microenvironment for adolescent Internet usage, where parental attitudes towards the Internet and mediation towards online behavior directly impact adolescents’ Internet adaptation. A critical usage orientation is advantageous for adolescents to accept parental guidance and reduce excessive or inappropriate Internet usage.

### 4.2. The Mediating Role of Parent–Child Relationships

This study has confirmed the mediating role of the parent–child relationship. Parent–child intimacy and conflict constitute two significant dimensions of the parent–child relationship [62] (pp. 188–213). During adolescence, the parent–child relationship often undergoes a transitional period characterized by heightened conflicts, decreased communication time, and diminished intimacy [30,63,64]. Active parental mediation facilitates communication and interaction between parents and children, fostering a warm, harmonious family atmosphere that is grounded in established rules. Such an environment benefits the parent–child relationship, as it provides emotional and intellectual support to the youth [65]. Thus, active mediation from parents can contribute to parent–child communication and reduce parent–child conflicts arising from Internet use during adolescence. Consequently, adolescents are less inclined to seek compensation through online activities. Furthermore, a harmonious parent–child relationship plays a crucial role in fostering adolescents’ recognition and acceptance of parental active mediation, thereby mitigating online risk behaviors [18,66]. The current study provides additional validation for the association between these two variables.

Drawing from the ecological techno-microsystem theory, it is evident that information technology and media have significantly altered the dynamics of interpersonal interaction and communication within families. These technological advancements provide family members with the opportunity to enhance their bonds, offsetting the limitations imposed by offline communication. Consequently, ordinary adolescents experience enhanced feelings of connection and subjective well-being [67,68]. Within the framework of active parental mediation, adolescents are provided with the opportunity to express their perspectives on digital technology, thereby reinforcing the notion of “democratic families” [69]. Therefore, it can be argued that active parental mediation serves a pivotal role, not only in employing specific mediation techniques, but also in fostering an atmosphere characterized by respect, support, and a resilient parent–child relationship. Adolescents perceive these relationships, which aid in the development of positive emotional and cognitive patterns, the establishment of stable offline interpersonal connections, the facilitation of effective communication, and the enhancement of their ability to cope with diverse risks and challenges in life, including those associated with Internet usage.

### 4.3. The Multiple Mediating Model

The present study validates the multiple chain mediating effects of two variables, namely the parent–child relationship and hiding online behavior, in the association between active parental mediation and PIU. Notably, a significant negative correlation was observed between the parent–child relationship and hiding online behavior, indicating that the mediating variables exhibited sequential characteristics, forming a chain of mediators. Thus, it can be inferred that the multiple mediation observed in this study follows a chain mediation model [51]. In particular, active parental mediation exerted a significant negative influence on adolescents’ tendency to hide their online behaviors from parents. This effect was facilitated through the positive impact it had on the parent–child relationship, ultimately resulting in a decrease in the likelihood of PIU.

Given that adolescents’ concealment of online behavior from parents essentially involves the act of secrecy in Internet use, Metzger et al. (2021) [37] incorporated risky online activities into the construct of adolescents keeping secrets from parents. Psychological reactance theory (PRT) provides a theoretical framework that elucidates the potential factors driving such defiant behavior. According to the PRT, individuals experience a state of aversion known as psychological impedance after being exposed to a threat to their freedom [70]. PRT further suggests that when individuals perceive a threat to their freedom, they are motivated to safeguard their freedom of choice and work towards restoring it (Brehm, 1966) [71]. The psychological reactance theory of adolescents towards parents’ hiding online behavior is manifested in adolescent group online behavior. When parents adopt a democratic guiding style, which involves proactive regulation, parent–child relationships become harmonious. Adolescents are able to express their views and demands regarding Internet use and receive timely feedback from their parents. Their need for autonomy is satisfied, reducing the likelihood of psychological reactance and actions such as hiding online behavior as a form of resistance. The possibility of seeking and guarding freedom is also minimized, thereby reducing the occurrence of problematic Internet use and achieving the educational effectiveness of proactive parental regulation. Our findings also suggests that parent–child interaction and the overall quality of the parent–child relationship, which constitute fundamental elements of the family environment, exert a substantial influence on adolescents’ inclination towards secrecy, the same with traditional secrecy behaviors [41].

This study establishes a parallel between hiding online behavior from parents and keeping secrets from them, as both behaviors share developmental characteristics and underlying factors. Previous research has shown that individuals who have strained relationships with their parents are more inclined to withhold information from them [38]. Consistent with these findings, the current study observed a similar association between the parent–child relationship and the act of hiding online behavior from parents. A positive parent–child relationship fosters a sense of respect and support, thereby encouraging adolescents to engage in self-disclosure and offline activities with their parents. This, in turn, reduces the risk of excessive Internet reliance in terms of both time and emotion, consequently diminishing the likelihood of concealing online behavior. It is worth noting that adolescent secrecy from parents is associated with externalizing problems [72]. Similar to conventional secrecy, hiding online behavior from parents reflects an individual’s personal awareness and information management approach [73]. Hiding online behavior serves as a response mechanism employed by adolescents to evade parental monitoring, inadvertently fostering an environment conducive to problematic Internet usage. Conversely, parents may also inaccurately assess the extent of their teenagers’ online presence and activities due to their adolescents’ tendency to hide online behavior [36]. Moreover, both hiding online behavior and PIU are strongly linked to an individual’s self-regulation abilities, with the latter being a crucial personal factor influencing adolescents’ susceptibility to PIU [3,74]. Hiding online behavior is characterized not only by its content of confidentiality but also by the ensuing consequences stemming from individual psycho-cognitive traits, which contribute to developing a dependency on Internet.

Hiding online behavior from parents can be viewed as a behavioral response stemming from psychological reactance. According to the PRT, when individuals perceive a threat to their freedom, they are motivated to safeguard their freedom of choice and work towards restoring it [71]. During adolescence, individuals have an increased need for autonomy. Active parental mediation is a form of a democratic parenting style that aligns with the psychological development of teenagers. Parents who utilize active mediation not only provide teenagers with greater opportunities to express their opinions but also grant them the freedom to use the Internet. Consequently, this form of parental mediation may reduce the likelihood of adolescents’ perception of freedom being threatened. As a result, teenagers are less likely to experience psychological reactance. The possibility of taking specific actions, such as hiding online behavior to regain freedom, is also diminished. Negative emotional reactions and psychological impedance serve as mediating factors in the association between parental psychological control and adolescent internalizing and externalizing behavioral problems [75]. Cultivating open communication between parents and teenagers can alleviate the psychological impedance experienced by adolescents, thereby facilitating increased expression towards parents and a reduction in secrecy.

Hiding online behavior, in isolation, does not serve as a mediating factor in the relationship between active parental mediation and PIU. This phenomenon can be elucidated through the definition of active mediation, which pertains to parents’ role in guiding their children to use media appropriately, rather than addressing teenagers’ concealment of online behavior. This distinction is also evident in the measurement items employed.

Furthermore, a masking effect was observed concerning the direct association between active parental mediation and problematic online use among adolescents. Despite the significant simple correlation, the direct link between active parental mediation and adolescents’ PIU did not retain its significance when parent–child relationships and hiding online behavior were taken into account. This indicates that the mediating variables of parent–child relationships and adolescent concealment of online behavior from parents were found to have inverse and positive correlations with adolescent PIU, respectively, resulting in the nullification of these two effects [76].

### 4.4. Theoretical and Practical Implications

Theoretically, this study aims to contribute to a novel wave of research on secrecy within adolescent–parent relationships. It recognizes that each individual and family is a distinct dynamic system with regards to information management, thereby highlighting that each adolescent responds to their parents in a personalized manner [77]. This study is an outcome of acknowledging the distinctive dynamic system inherent in parent–child communication. It underscores the significance of comprehensively examining the perception of the effectiveness of active parental mediation within the wider framework of the parent–child relationship, the adolescent’s response to parental mediation, and further validates the application of theories pertaining to parental confidentiality in comprehending adolescents’ hiding online behavior from their parents, although parental confidentiality is connected to autonomy and individuation, exhibits positive effects to a certain extent [78].

In practice, this study highlights the presence of adolescents’ hiding online behaviors that are not easily discernible. By illustrating these hidden behaviors, it sheds light on the challenges that parental mediation may encounter in real-world contexts and suggests potential avenues for resolution. One notable obstacle faced in parental mediation is identified as follows: adolescents hiding their online behavior due to their desire for independence and freedom from parental authority as they transition into adolescence. Simultaneously, they anticipate that excessive Internet use or engaging in certain online activities will result in parental discipline and reprimand. Consequently, opting to hide their online activities becomes an autonomous decision based on weighing these factors, bolstered by the technological advantage that often enables successful concealment. As a result, parents find it challenging to detect their children’s online activities. The primary and efficacious approach to overcome this obstacle involves parents fostering a strong parent–child relationship through activities such as explaining and discussing media content and usage. Additionally, parents should guide their children in the proper use of media. By cultivating a positive parent–child relationship, various benefits can be achieved, including the enhancement of adolescents’ subjective well-being, bolstering their stress coping abilities, reducing loneliness, stimulating their interest in communicating with parents, and diminishing their reliance on the Internet. It is imperative to recognize the practical significance of adolescents’ proactive approach in dealing with parental mediation and to heighten our vigilance accordingly.

### 4.5. Limitations and Prospects

Although this study meets the requirements of academic research in general and the research ideas are clear, there are still some shortcomings.

Regarding the data collected on adolescent PIU, there appeared to be a tendency towards centralized responses during participant completion. This resulted in a limited standard deviation of the data, which has the potential to detrimentally impact data quality. In terms of parental active mediation, this study solely relied on self-reports provided by adolescents. However, incorporating parental reports alongside adolescent reports could offer a more comprehensive and accurate representation of the extent of active parental mediation. Furthermore, as a cross-sectional study, this research primarily establishes correlations among variables. However, in order to validate the findings and provide a deeper understanding of the causal relationships, it is recommended to conduct follow-up studies using longitudinal research methods. Additionally, due to the focus on one cultural (Chinese) context, another limitation of our research is the potential lack of generalizability, the findings are difficult to generalize to a broader cultural context.

This study aims to elucidate the association between active parental mediation and PIU among adolescents. We highlight two significant mediators: (1) hiding online behavior from parents, which serves as a nascent but crucial negative mediator, and (2) the parent–child relationship, which has been extensively validated as a mediator. By constructing a fully mediated model, we aim to provide a comprehensive framework for better comprehending the intricate relationship between active parental mediation and adolescent PIU. Future research should focus on examining how the Internet can be effectively utilized within the family setting to enhance the parent–child relationship and foster a warm and harmonious familial atmosphere. This entails establishing consistent guidelines and promoting critical thinking skills, which can help reduce adolescents’ inclination to hide their online activities. By exploring these aspects, we can potentially mitigate negative online behaviors among adolescents.

## 5. Conclusions

A significant and inverse correlation was observed between active parental mediation and adolescent PIU, highlighting the preventive and educational benefits of active parental mediation in mitigating offspring PIU.

The parent–child relationship was found to play a mediating role in the association between active parental mediation and adolescent PIU, illustrating the underlying mechanism by which parental active mediation influences PIU.

Adolescents’ tendency to hide their online behavior from parents exhibited a positive correlation with PIU, while demonstrating a significant negative association with the quality of the parent–child relationship. This pattern of behavior indicates a propensity for risk-taking.

Both the act of hiding online behavior and the state of the parent–child relationship played a complete mediating role in the relationship between active parental mediation and adolescent PIU. This highlights an alternative mechanism through which active parental mediation influences PIU.

## Figures and Tables

**Table 1:** Descriptive statistics and correlation analysis of variables.

Variables	M	SD	1	2	3	4
Active parental mediation	4.710	0.988	1			
Parent–child relationship	2.445	0.363	0.422 ***	1		
Hiding online behavior	1.781	0.751	−0.225 ***	−0.345 ***	1	
Problematic Internet use	2.306	0.038	−0.225 ***	−0.417 ***	0.545 ***	1

Note: *n* = 539, *** *p* < 0.001.

**Table 2:** Regression analysis of the relationship among variables.

Regression Equations Coefficients		Overall Fit Index			Significance of Regression Coefficients	
Result Variables	Predictor Variables	*R*	*R* ^2^	*F*	*β*	*t*
PIU	APM				−0.225	−5.313 ***
PCR	Gender	0.424	0.180	39.033	−0.191	−2.411 *
	Age				0.000	0.005
	PCR				0.426	10.735 ***
HOB	Gender	0.383	0.146	22.284	−0.034	−0.420
	Age				0.169	4.197 ***
	APM				−0.096	−2.141 *
	PCR				−0.302	−6.850 ***
PIU	Gender	0.610	0.372	63.107	−0.066	−0.953
	Age				0.090	2.566 *
	APM				−0.015	−0.393
	PCR				−0.252	−6.365 **
	HOB				0.454	12.228 ***

Note: PIU—Problematic Internet use, APM—Active parental mediation, PCR—Parent–child relationships, HOB—Hiding online behavior; * *p* < 0.05, ** *p* < 0.01, *** *p* < 0.001.Variables other than gender were standardized.

**Table 3:** Bootstrap 95% confidence intervals for mediated effect pathways.

Intermediary Pathway	Effect Value	95% Confidence Interval		Relative Mediating
		Lower Limit	Upper Limit	Effect ^1^
APM → PCR → PIU	−0.107	−0.151	−0.069	47.795%
APM → HOB → PIU	−0.044	−0.085	0.004	
APM → PCR → HOB → PIU	−0.059	−0.092	−0.033	26.102%

Note: PIU—Problematic Internet use, APM—Active parental mediation, PCR—Parent–child relationships, HOB—Hiding online behavior. ^1^ The ratio of indirect effects to total effects.

## Data Availability

The raw data for this article will be made available by the authors upon reasonable request.
